# Association of the methylenetetrahydrofolate reductase (*MTHFR*) C677T polymorphism with primary glaucoma in Saudi population

**DOI:** 10.1186/s12886-016-0337-7

**Published:** 2016-09-01

**Authors:** Hamoud Al-Shahrani, Najwa Al-Dabbagh, Nourah Al-Dohayan, Misbahul Arfin, Mohammad Al-Asmari, Sadaf Rizvi, Abdulrahman Al-Asmari

**Affiliations:** 1 Department of Ophthalmology, Prince Sultan Military Medical City, Riyadh, Saudi Arabia; 2 Research Centre, Prince Sultan Military Medical City, P.O. Box 7897, Riyadh, 11159 Saudi Arabia

**Keywords:** *MTHFR*, Glaucoma, Polymorphism, Genotyping, Genetics, Saudi

## Abstract

**Background:**

Methylenetetrahydrofolate reductase (MTHFR), a critical enzyme in folate metabolism is involved in DNA synthesis, DNA repair and DNA methylation. The functional polymorphism of *MTHFR gene*, C677T has been shown to impact various diseases and implicated as a risk factor for the development of various neurodegenerative disorders including glaucoma.

**Methods:**

We investigated *MTHFR C677T* genotypes and alleles frequencies in primary glaucoma [primary open angle glaucoma (POAG) and primary angle closure glaucoma (PACG)] patients and matched healthy controls in a case-control study. Two hundred ten primary glaucoma cases were studied for *MTHFR C677T* polymorphism and compared with 280 controls taken from the healthy population, employing the polymerase chain reaction-restriction fragment length polymorphism technique (PCR-RFLP). The *MTHFR* gene was amplified using specific primers. The PCR products (294 bp) was subsequently digested with HinfI (New England Biolabs) at 37 °C for 12 h, separated by electrophoresis on 2 % agarose gels, and visualized with ethidium bromide staining. The restriction digestion yielded 168 and 126 bp fragments for TT, 294, 168 and 126 bp fragments for CT and undigested PCR product 294 bp indicating CC genotype.

**Results:**

We found the frequency of the genotypes and alleles of *MTHFR C677T* differ significantly between cases and controls. The frequencies of allele T and genotype CT were significantly higher while the frequencies of allele C and genotype CC were lower in primary glaucoma patients as compared to controls (*p* <0.05). Upon stratification of our results into POAG and PACG, significantly higher frequencies of allele T (19.44 %) and genotype CT (38.89 %) were found in POAG patients compared to controls (12.5 % and 25 % respectively). The frequencies of alleles and genotypes were almost similar in PACG and controls (*p* = 0.8).

**Conclusion:**

This study indicates that the allele T and genotype CT of *MTHFR* C677T polymorphism are significantly associated with POAG while allele C and CC genotype may be protective for it. We conclude that the *MTHFR* C677T polymorphism increases the risk for POAG development in Saudi population and can be a genetic marker however, further studies are needed with multiple-ethnic populations affected with POAG to strengthen these findings.

## Background

Glaucoma is the second leading cause of blindness worldwide. It is a relatively common eye disease characterized by the pathological loss of retinal ganglion cells resulting into progressive loss of sight and related changes in the retinal nerve fiber layer and optic nerve head [1]. Glaucoma affects 70 million people worldwide and by the year 2020, this number is estimated to rise to around 79.6 million [2]. Glaucoma can be classified as primary or secondary based on the etiology and aqueous humor dynamics [3]. Primary glaucoma (PG) is one of the most common optic neuropathies and further classified as primary open angle glaucoma (POAG) and primary angle closure glaucoma (PACG) on the basis of gonioscopy (anterior chamber anatomy) and their specific etiology.

Increased levels of homocysteine in plasma, serum and tear have been found in patients with POAG, PACG and pseudoexfoliative glaucoma [4–11]. The methylenetetrahydrofolate reductase (*MTHFR*) C677T (rs1801133) is missense variation, located in exon of the *MTHFR* gene on chromosome 1 and also known as Ala222Val or A222V. *MTHFR* C677T polymorphism leads to an increase in thermolability and reduced activity of the MTHFR enzyme [12]. This reduction in enzymatic activity of MTHFR ultimately leads to the elevation of homocysteine levels in plasma [13]. MTHFR (EC1.5.1.20), being a crucial enzyme in the pathway, diverts folate content towards homocysteine remethylation. This enzyme catalyzes reduction of 5,10-methylenetetrahydrofolate to 5-methyltetrahydrofolate, which acts as methyl donor for remethylation of homocysteine to methionine [13].

The C677T polymorphism in the *MTHFR* gene causes reduction in the activity of a thermolabile form of MTHFR which is the most common genetic factor for moderate hyperhomocysteinemia. The prevalence of *MTHFR* C677T polymorphism has been reported to be high in POAG patients [4, 14, 15]. However, earlier reports on the association between *MTHFR* C677T polymorphism and glaucoma risk are inconclusive and conflicting [6, 7, 16–20]. Saudi population being a closed and isolated society with high rate of consanguinity (inbreeding) is ideal for such genetic association studies. So we decided to analyze any possible association between *MTFHR* C677T polymorphism and PG (POAG and PACG) in a cohort of Saudi population.

## Methods

### Subjects

The present study was undertaken to analyze 490 Saudi subjects for the frequency of allele and genotype of *MTHFR* C677T polymorphism. The sample size was calculated online (http://sampsize.sourceforge.net/iface/s3.html). Initially we recruited 300 sample each for patient and control group but after applying inclusion and exclusion criteria only 210 patients and 280 controls were found suitable for inclusion in the study. A total of 210 unrelated Saudi patients with PG including POAG (*n* = 144) and PACG (*n* = 66) were recruited from ophthalmology clinic of the Prince Sultan Military Medical City (PSMMC) Saudi Arabia. The demographic characteristics of patients and healthy controls are summarized in Table 1. Males (106) and females (104), aged 30-78 years (mean ± SD: 58 ± 14.4) constituted the patient group while 280 unrelated healthy Saudi subjects of same ethnicity, aged 30 - 78 years (mean ± SD: 56 ± 11.6) were recruited as controls. The PG patients were diagnosed on the basis of clinical observation as described earlier [21, 22]. Patients with confirmed diagnosis of PACG or POAG free from any other systemic and autoimmune diseases were selected for this study. General inclusion criteria included ability and willingness to provide informed consent, participants may be male or female of age not less than 30 years. The subjects with the diagnosis or history of any secondary glaucoma, ocular trauma or significant use of systemic or ocular glucocorticoids were excluded.

**Table 1:** Demographic characteristics of patient and controls

Primary glaucoma patients (210)	
Age (years)	
Range	30-78
Mean ± SD	58 ± 14.4
Gender (%)	
Male	106 (50.48)
Female	104 (49.52)
Family history	
Without	158 (75.24)
With	52 (24.76)
Bilateral presentation	
Yes	143 (68.09)
No	67 (31.91)
Primary open angle glaucoma (POAG) (144)	
Age (years)	
Range	30-78
Mean	60 ± 12.2
Gender (%)	
Male	81 (56.25)
Female	63 (43.75)
Primary angle closure glaucoma (PACG) (66)	
Age (years)	
Range	32-74
Mean	56 ± 15.6
Gender (%)	
Male	25 (37.88)
Female	41 (62.12)
Healthy Controls (280)	
Age (years)	
Range	30-78
Mean	56 ± 11.6
Gender (%)	
Male	145 (51.79)
Female	135 (48.21)

Patients with history of other ocular or neurologic disease or surgery that causes visual loss, systemic diseases [lupus, Graves, cancer (within the last 5 years), AIDS, other] that cause visual loss, or with history of amblyopia were also excluded. Patients with signs of intracranial disease were also excluded. Professionally trained glaucoma specialists reviewed medical records for all cases and control subjects. Additionally all the control subjects were examined by an ophthalmologist for documentation of cup-to-disc ratio, and any history of glaucoma, elevated IOP, or optic nerve abnormality. Controls with history of any systemic or autoimmune disease were excluded.

The blood samples were collected and stored at -80 °C. DNA was extracted using standard technique. The study protocol was approved by the ethical committee of the Prince Sultan Military Medical City (PSMMC) Riyadh, Saudi Arabia (via # 318/2013 dated 24.4.2013) and written informed consent was obtained from all participants.

### Genotyping

Detection of the genotypes/alleles of *MTHFR C*677T polymorphism was performed with PCR amplification using a set of primers. The PCR was performed following the protocol as described elsewhere [6]. The PCR products (294 bp) were subsequently digested with HinfI at 37 °C for 12 h, separated by electrophoresis on 2 % agarose gels, and visualized with ethidium bromide staining. The restriction digestion yields 168 and 126 bp fragments for TT, 294, 168 and 126 bp fragments for CT and undigested PCR product 294 bp indicating CC genotype. For quality control the genotyping was repeated for 25 % of the random blind samples and results were compared. The positive and negative controls were also used in the PCR.

### Statistical analysis

The genotyping results were analyzed and frequencies of alleles and genotypes were calculated. Hardy-Weinberg Equilibrium Calculator for 2 Alleles (http//www.had2know.com/academics/hardy-weinberg-equilibrium-calculator-2alleles.html) was used to calculate Hardy-Weinberg equilibrium. The differences in the frequencies of alleles and genotypes between patients and controls were analyzed by the Fisher’s exact test using the CalcFisher software [23]. *P* values ≤ 0.05 were considered significant. The strength of the association of disease with respect to a particular allele/genotype is expressed by odd ratio interpreted as *relative risk* (RR) following the Woolf’s method as out lined by Schallreuter et al. [24]. The hypothetical genetic component of the disease, etiologic fraction (EF) and the hypothetical protective effect of one specific allele/ genotype for the disease, preventive fraction (PF) were calculated following formula given by Svejgaard et al. [25] as described in details in our earlier publication [22].

## Results

The results of the genotypes and alleles distribution of *MTHFR* C677T polymorphism in PG and controls are summarized in Tables 2, 3, 4, 5. The single nucleotide polymorphism in the *MTHFR* gene (rs1801133) was in Hardy–Weinberg equilibrium for patients while not in controls possibly due to high consanguinity. The frequency of CT genotype was significantly higher (34.76 % vs 25 %, *p* = 0.02) while the frequency of CC genotype was lower (65.24 % vs 75 %, *p* = 0.02) in primary glaucoma patients as compared to controls. Genotype TT was absent both in the patients and controls groups.

**Table 2:** Genotype and allele frequencies of MTHFR (C677T) variants in primary glaucoma patients and matched controls

Genotype/allele	PG (*N* = 210)		Control (*N* = 280)		*p*-value	*RR*	*EF* ^b^ */PF*
	N	%	N	%			
CC	137	65.24	210	75.00	0.02^a^	0.63	0.194
CT	73	34.76	70	25.00	0.02^a^	1.60	0.191^b^
TT	0	0	0	-	-	-	-
C-allele	347	82.62	490	87.50	0.03^a^	0.68	0.152
T-allele	73	17.38	70	12.50	0.03^a^	1.47	0.163^b^

*N* number of subjects, *EF* etiological fraction, *PF* preventive fraction

^a^statistically significant

^b^data for EF

**Table 3:** Genotype and allele frequencies of MTHFR (C677T) variants in male and female primary glaucoma patients

Genotype/allele	Male (106)		Female (104)		*p*-value
	*N*	%	*N*	%	
CC	67	63.21	70	67.31	0.56
CT	39	36.79	34	32.69	0.56
TT	0	-	0	-	-
C-allele	173	81.60	174	83.65	0.60
T-allele	39	18.40	34	16.35	0.60

*N* number of subjects

**Table 4:** Genotype and allele frequencies of MTHFR (C677T) variants in POAG patients and matched controls

Genotype/allele	POAG (*N* = 144)		Control (280)		*P*- value	RR	EF^b^/PF
	N	%	N	%			
CC	88	61.11	210	75.00	0.003^a^	0.52	0.214
CT	56	38.89	70	25.00	0.003^a^	1.91	0.211^b^
TT	0	-	0	-	-	-	-
C-allele	232	80.56	490	87.50	0.008^a^	0.59	0.182
T-allele	56	19.44	70	12.50	0.008^a^	1.69	0.181^b^

*N* number of subjects, *EF* etiological fraction, *PF* preventive fraction

^a^statistically significant

^b^data for EF

**Table 5:** Genotype and allele frequencies of MTHFR (C677T) variants in PACG patients and matched controls

Genotype/allele	PACG (*N* = 66)		Control (280)		*P*- value	RR	EF^a^/PF
	N	%	N	%			
CC	49	74.24	210	75.00	0.87	0.961	0.007
CT	17	25.76	70	25.00	0.87	1.041	0.007^a^
TT	0	-	0	-	-		-
C-allele	115	87.12	490	87.50	0.88	0.966	0.171
T-allele	17	12.88	70	12.50	0.88	1.0347	0.170^a^

*N* number of subjects, *EF* etiological fraction, *PF* preventive fraction

^a^data for EF

Frequency of allele T was found to be significantly higher while that of allele C was lower in PG patient than controls (*P* = 0.03). Upon stratification of PG patients gender vise, no significant difference was found in distribution of alleles and genotypes of *MTHFR* C677T polymorphisms between male and female patients (Table 3).

The genotyping results after stratification into POAG and PACG and comparison with that of control group are summarized in Tables 4 and 5. The frequencies of genotype CT and allele T were significantly higher (*p* = 0.003, *p* = 0.008 respectively) in POAG as compared to controls. While the frequencies of genotype CC and allele C were significantly lower in POAG as compared to controls (Table 4). On the other hand there was no significant difference in the frequencies of both the alleles and genotype between PACG and controls (*p* = 0.88). The frequency distribution of alleles and genotypes of *MTHFR* C677T in POAG and PACG shows similar pattern and no significant difference could be noticed in the frequencies of alleles and genotypes between PACG and POAG. The results of repeated genotyping for 25 % of the random blind samples on comparison with earlier results indicted 100 % success rate. Genotype distribution of *MTHFR* C677T polymorphism in different healthy population worldwide is summarized in Table 6 which clearly indicates ethnic variations.

**Table 6:** Genotype distribution of MTHFR C677T polymorphism in different healthy populations

Population/group studied	Sample number	Genotype frequencies % CC CT TT			Reference
Saudis	280	75	25	0	Present study
American Whites	300	47.33	42.00	10.67	[45]
American Blacks	298	77.52	19.80	2.68	[45]
Australian	288	50.69	41.32	7.99	[45]
Austrian Caucasian	211	49.76	40.76	9.48	[16]
British Caucasian	58	46.55	46.55	6.90	[46]
Canadian whites	240	56.67	37.50	5.83	[45]
Chinese	381	57.74	30.71	11.55	[47]
Chinese	878	23	49.1	27.9	[48]
Colombian	152	34.45	52.55	13.0	[49]
Dutch	188	51.60	42.02	6.38	[45]
Egyptian	149	76.50	20.10	3.40	[50]
Finish	545	53.76	42.20	4.04	[45]
French	178	40.45	47.75	11.80	[45]
German	71	63	34	3	[14]
Greek	135	30	53.85	16.15	[51]
Han Chinese North	643	31.26	48.99	19.75	[45]
Han Chinese South	430	29	53	8.1	[45]
Indian	173	79.19	19.65	1.16	[15]
Indian	70	71.43	25.71	2.86	[52]
Indian	100	69	26	5	[53]
Iranian	90	58.89	36.67	4.44	[29]
Iranian females	116	54.3	37.1	8.6	[54]
Israeli	210	57.14	34.29	8.57	[45]
Italian whites	385	33.25	51.43	15.32	[45]
Japanese	106	45.3	36.8	17.9	[28]
Jordanian	150	52.77	34.53	12.70	[55]
Kashmiri	160	75.6	16.9	7.5	[56]
Korean	100	31	50	19	[20]
Mexican Nahuas	135	5.18	42.97	51.85	[57]
Mexican Mixtecas	124	9.7	40.3	50	[57]
Mexican Mestizas	196	16.8	44.4	38.8	[57]
Pakistani Pathan	70	76	23	1	[6]
Pakistani Punjabi	73	66	34	0	
Pakistani	70	81.43	18.57	0	[58]
Russian	587	53.15	39.86	6.99	[45]
Spanish whites	601	44.09	44.09	11.82	[45]
Turkish	100	51	43	6	[59]
Turkish	212	97.6	2.4	0	[60]

## Discussion

Results of the present study revealed significant differences in the frequencies of alleles and genotypes of *MTHFR* C677T polymorphism between PG patients and controls (Table 1). The significantly higher frequencies of genotype CT and allele T in PG patients as compared to controls indicated that the genotype CT and allele T are associated with susceptibility to the PG (RR = 1.60, EF = 0.19 and RR = 1.47, EF = 0.16 respectively). The higher frequency of the T allele and predominance of CT genotypes in PG patients in comparison with matched controls suggested that allele T carriers are at a higher risk of developing PG in Saudi population. The genotype CC and allele C being higher in controls indicated their protective nature for PG (RR = 0.63, PF = 0.19 and RR = 0.68, PF = 0.15 respectively). In gender wise analysis, the genotype and allelic frequencies did not differ significantly between males and females indicating that the distribution of alleles/ genotypes of *MTHFR* C677T is not affected by any specific gender.

Further, stratification of results into PAOG and PACG, revealed that the frequency distribution of alleles and genotypes differed in two types of glaucoma. The higher frequency of CT genotype and allele T in POAG indicated that the genotype CT and allele T of *MTHFR* C677T polymorphism might be associated with susceptibility risk of POAG (RR = 1.91, EF = 0.211) while the decreased frequency of genotype CC in POAG as compared to controls indicated that genotype CC may be resistant to POAG in Saudis. Our results are in agreement with the earlier findings which reported similar association between *MTHFR* C677T polymorphism and POAG in German and Indian patients [4, 14, 15]. Similarly *MTHFR* C677T polymorphism has also been reported as a genetic risk factor of normal tension glaucoma (NTG) in the Korean population [20].

A significant associations between MTHFR C677T polymorphism and POAG was suggested recently and a meta-analysis indicated that the T allele or TT genotype might increase the risk of POAG [26]. However, the need for further studies with POAG patients using large sample-size from multiple ethnicity has been emphasized to reach any definite conclusion.

On the other hand, several other studies suggested that *MTHFR* C677T polymorphism itself is not a major risk factor [10, 27] as these studies could not find any significant association with susceptibility risk of POAG in Japanese [28], Austrian [16], Swedish [18], Pakistani [6], Iranian [29], Mexican [30] and Greek populations [13].

Our results also indicated a lack of association between the *MTHFR* C677T polymorphism and PACG in Saudi population as the frequencies of allele and genotypes were almost similar in PACG and control subjects (*p* = 0.88, Table 4). Similar to our findings, no association of *MTHFR* C677T polymorphism has been found with the susceptibility to PACG in Nepalese and Australian [31], and Indian [15]. Contrary to these findings one report from Pakistan indicates that the *MTHFR* C677T polymorphism is associated with PACG. From the available data it is evident that the prevalence of *MTHFR* C677T polymorphism varies in different ethnic healthy populations and also among the different types of glaucoma. The frequencies of TT genotype vary from 0 to 28 %, except in Mexican population where it is quite high (52 %), those of CT genotype from (2.4-53 %) and CC genotype from 23-97 % in various populations worldwide (Table 6). Similar to our finding two reports one each from Pakistan and Turkey indicates absence of TT genotype in healthy cohort. The ethnic variations in the distribution of alleles and genotype of *MTHFR* polymorphism in various populations and types of glaucoma might have lead to differences in the findings.

The result of this study also showed complete absence of homozygous genotype (TT) in our control and patient groups. The frequency of heterozygous genotype CT was 25 % and that of homozygous CC 75 % in Saudi healthy population. Global studies on distribution of genotypes of *MTHFR* C677T in healthy population have shown highly significant variations in genotype frequencies of TT, CT and CC (Table 6). The reason for the dissimilar findings in different populations is not clear and may reflect ethical differences/ geographical variations in the genotype frequencies. Chiras et al. [32] also reported variable frequency especially of genotype TT in populations from different geographic areas. The highest frequency TT genotype of *MTHFR* C677T was found in Mexico and the lowest in middle east, African countries, India and Pakistan. Therefore, it is important to pay special attention on genetic association studies in ethnic groups like Saudi Arabians that have remained isolated from the rest of the world populations and has retained their own cultural characteristics, lifestyle, and traditions.

The *MTHFR* C677T polymorphism influences homocysteine metabolism and hyperhomocysteinemia has been reported in glaucoma patients [4–11]. The *MTHFR* C677T polymorphism causes an alanine > valine amino acid substitution, which influences the activity of MTHFR enzyme. As a results of this polymorphism 55–65 % loss in enzyme activity has been observed in individuals who are homozygous for the mutation (TT carriers), while in the case of heterozygotes (CT carriers) there is a 25 % loss in activity compared to CC homozygous normal individuals [13, 33]. This reduced activity of MTHFR leads to hyperhomocysteinemia. The significantly higher frequency of genotype CT in our POAG patients as compared to controls might have resulted in moderate hyperhomocysteinaemia. Although the exact biologic role of hyperhomocysteinemia in glaucoma is not clear yet, however it has been linked to other vascular diseases [34] and reported to cause dysregulation of matrix metalloproteinases and their inhibitors [35]. The dysregulation of matrix metalloproteinases has been implicated in the pathogenesis of glaucoma [36]. Homocysteine can also induce vascular injuries [37], alterations in the extracellular matrix [38], and neuronal cell death by inducing apoptosis or excitotoxicity [39, 40]. Moreover, hyperhomocysteinemia has been shown to be involved in the structural remodeling of connective tissues [41].

Hyperhomocysteinemia induced by the *MTHFR* C677T polymorphism has been linked with neuronal cell death of retinal ganglion cells (RGC), a characteristic feature of glaucoma [15]. Despite the clinical heterogeneity, all forms of glaucoma ultimately result in death of retinal ganglion cells (RGC) in the optic nerve. Homocysteine has been reported to induce apoptotic cell death of RGCs [15, 40] and also exerts gliotoxic effects, thus representing a risk factor for POAG [42]. Apoptotic death of RGCs in glaucoma has been supported by various earlier molecular studies [43, 44].

Our results indicate a significant association of *MTHFR* C677T with POAG but not with PACG in Saudi population suggesting that the CT genotype or T allele predisposes individuals toward POAG. In PACG, though the angle architecture has been attributed for the initiation of the disease however, other factors may also be involved. On the other hand in POAG, in spite of normal anterior angle structures, patients develop glaucomatous optic atrophy, indicating that multiple factors including genetic ones may contribute towards its pathogenesis. This study showed that *MTHFR* C677T might be one of those contributing genetic factors for POAG in Saudi population.

This is the first report suggesting a significant association of *MTHFR* C677T with POAG in a subset of Saudi population. On the other hand lack of any association between PACG and *MTHFR* C677T indicated that factors other than *MTHFR* C677T could be important players in susceptibility to PACG in our samples and future studies involving different genes should be designed. Present study suggested that the T allele of C677T imposed increased risk for POAG in Saudi population, supporting the hyperhomocysteinemia mediated death of RGC in POAG. Our study also indicated that the distribution of frequencies of genotypes and alleles of *MTHFR* C677T polymorphism is not affected by gender of the patients (Table 2). However, further studies should be conducted in a larger cohort of males and females affected with POAG to confirm it.

## Conclusion

In conclusion the *MTHFR* C677T polymorphism is significantly associated with susceptibility to POAG in our population and can be a possible genetic marker for evaluating the risk. however, further studies with multiple-ethnic populations affected with POAG will strengthen these findings.

## Abbreviations

EF: Etiologic fraction
MTHFR: Methylenetetrahydrofolate reductase
PACG: Primary angle closure glaucoma
PCR-RFLP: Polymerase chain reaction- restriction fragment length polymorphism
PF: Preventive fraction
PG: Primary glaucoma
POAG: Primary open angle glaucoma
PSMMC: Prince Sultan Military Medical City
RR: Relative risk
SNP: Single nucleotide polymorphism
